# Visibility and Social Recognition as Psychosocial Work Environment Factors among Cleaners in A Multi-Ethnic Workplace Intervention

**DOI:** 10.3390/ijerph10010085

**Published:** 2012-12-24

**Authors:** Kirsten Hviid, Louise Hardman Smith, Karen Bo Frydendall, Mari-Ann Flyvholm

**Affiliations:** 1 REMESO, Linköping University, Holmentorget 10, SE-1601 74 Norrköping, Sweden E-Mail: kirsten.hviid@liu.se; 2 The National Research Centre for the Working Environment, Lersø Parkallé 105, DK-2100 Copenhagen, Denmark; E-Mails: lhs@nrcwe.dk (L.H.S.); kbf@nrcwe.dk (K.B.F.)

**Keywords:** immigrants, new labour migrants, cleaning industry, psychosocial work environment, visibility

## Abstract

This article focuses on the psychosocial work environment of immigrant cleaners at a Danish workplace. Today, many cleaners working in Danish cleaning jobs are women from the established immigrant communities, but also labour migrants from the newer EU member states have found their way to the cleaning industry. Studies have drawn attention to immigrants’ low position in the cleaning industry and their increased risk of work injuries. This article is based on a case study of an intervention called “Make a Difference” designed to improve the work environment among cleaners at a multi-ethnic workplace. We used semi-structured interviews, photo logs, observation and participation to investigate how the cleaners experienced their work environment. The cleaners reported an overload of heavy work, related to the concept of a classroom’s “readiness for cleaning”, and they expressed strained social relations and communication in addition to a lack of social recognition and invisibility at the workplace, a school. We analysed these psychosocial work environmental problems by investigating the different forms of social relationships and communication within the group of cleaners, and between the cleaners and the teachers and pupils at the school. Moreover, we discussed why the intervention, based on training of language and cleaning skills and social interaction, only partially improved the cleaners’ psychosocial work environment problems. In this article, we argue that social divisions based on ethnicity between the new and the established group of cleaners, combined with their marginal position and poor work organisation at the school, reinforced the cleaners’ experiences of psychosocial work environment problems. This article suggests that increased effort towards social inclusion at work and improved work organisation, especially for the new labour migrants from newer EU-countries, should be considered.

## 1. Introduction

Over the past two decades the service industry has grown radically in size and function partly due to periods of economic expansion [1]. The service industry has been subject to global competition and outsourcing of services requiring constant flexibility of the service producers in order to provide service products for new specialized demands. Consequently the cleaning industry has become more differentiated in the types of work; traditional contra managerial work, separating skilled and unskilled job functions, increasing the number of full-time jobs as well as the workload with larger areas to be cleaned at a faster pace. Outsourcing of cleaning services also means that workplaces are being separated physically from the cleaning companies. Often the cleaners are working at odd hours and they are becoming more isolated and limited in their social contact to managers, colleagues, users or customers [2]. Changes in type of employees in relation to gender, social, and especially ethnic backgrounds have taken place. In the past decade there has been a development with an increase in cleaners with immigrant backgrounds, men and women, unskilled and skilled, long and short time residents, and a decline in native born cleaners. Studies have concluded that labour migrants are mainly recruited into the service industry because their flexibility and mobility are suitable for the types of work in cleaning involving shifts between different working hours and sites [1,2]. Different aspects of immigrants working as cleaners in the cleaning services have been studied, such as ethnicity, gender, and segregation [2,3]. These studies show that ethnic minorities, males as well as females, are recruited to work in unskilled jobs in the cleaning industry at the labour markets in Western societies, and they often work at ethnically segregated workplaces. This economic, organisational, and social development in the service industry and cleaning industry has taken place in Denmark in the past decade and especially after the EU-enlargement in 2004. 

### 1.1. Cleaners with Immigrant Background in Denmark

The immigrants in Denmark arriving during the 1980s and 1990s did not all enter as migrant workers in contrast to many other Western countries, e.g., Germany [4], most entered as a consequence of family reunification or asylum. In 2001, after a change of government, a new migration policy was introduced in Denmark, restricting family reunification and asylum immigration. In addition, a policy, “New Chance for All”, was implemented by the Danish government in 2008 with the purpose to employ citizens who were currently outside the labour market, including immigrants. Many immigrants got employment in the cleaning industry in which there was a shortage of labour [2].

Furthermore, after 2004 and especially in 2007 with the EU-enlargements, many new labour migrants arrived from the 10 new EU countries, the Czech Republic, Hungary, Poland, Slovakia, Slovenia, Latvia, Lithuania, Estonia, Romania, and Bulgaria. Many of these new labour migrants now work in cleaning jobs.

The percentages of all employees at the labour market and in the service sector according to origin (non-Western, Western, or Danish), gender (male or female) and age (between 16–64 years) in 2009 are listed in Table 1. The data in Table 1 show that of the three groups of employees, female immigrants from non-Western countries represent the highest percentage of employees in the service sector (including operational services) dominated by cleaning companies [5]. Statistics Denmark applies the definition of non-Western countries as: “All other countries not included in the EU, Scandinavia, USA, Canada, Australia, New Zealand, Andorra, Liechtenstein, Monaco, San Marino, Switzerland and the Vatican State”. A relatively high percentage of the non-Western male employees are also employed in this service sector. Western female and male employees have the second highest percentage of the total number of employees in the service industry. The Danish employees have the lowest percentage of employees of the total workforce in the service industry. The number confirms findings from other studies that the service industry is an important sector of employment especially for non-Western female and male immigrants, but also for Western female and male immigrants. 

**Table 1:** Employees in the service sector compared to all employees (ages 16–64 years) at the labour market according to origin and gender, 2009 [5].

Employees’ origin	Non-Western		Western		Danish	
	Male	Female	Male	Female	Male	Female
Total employees age 16–64 years	56,120	51,173	39,277	34,796	1,169,788	1,144,229
Employees in the service sector	6,398	9,109	3,024	3,584	43,282	45,769
Percentage of the employees in the service sector	11.4%	17.8%	7.7%	10.3%	3.7%	4.0%

There are only limited international and Danish studies on work injuries and sick leave among immigrants in the cleaning services [6,7]. However, some work injury research shows that employees with immigrant status have a higher risk of work injuries than the majority population within the same trade [8]. The incidences of work related accidents among “non-Danish workers” is on average 60% above the average for all notified work related accidents, although immigrant cleaners have a slightly lower incidence compared to the total incidence of accidents in the cleaning industry [9]. 

In a Danish study based on 500 Polish respondents living in Copenhagen, the Polish employees were six times more likely to be working in the cleaning services area than Danish employees and they reported more work overload and working at a faster rate than Danish employees [10]. The increased exposure of immigrants to work environmental health problems has been explained by the fact that labour migrants are often employed in high risk jobs and are unfamiliar with safety rules, partly because of poor Danish language skills. Few studies, however, have investigated how the labour migrants are integrated at work, in terms of instruction on work environment, safety, and cooperation as required by the Danish Working Environment Act [2]. 

This article will contribute with new insights into issues of the work environment among cleaners with immigrant backgrounds and efforts made to integrate immigrant groups at workplaces. It is based on a study of an intervention called “Make a Difference”, designed by the cleaning department of a Danish municipality to improve the psychosocial work environment of a group of cleaners with mainly immigrant background at a school to reduce high levels of sick leave. The purpose of the intervention was partly to raise the standard of cleaning and partly to improve the psychosocial work environment of the cleaners. They were offered training to improve their status as employees. In a study evaluating the impact of the intervention on the psychosocial work environment, the participating cleaners expressed particular problems at work, such as problems regarding the concept of a classroom’s “readiness for cleaning” (Section 3.3) [11]. The first aim of this study was to investigate the changing social processes during the intervention, and to explain its outcomes by analysing the work environmental problems, such as the “readiness for cleaning”, “social relations and communication” and “social recognition and respect”. The second aim was to understand how the social position and ethnicity of the immigrant cleaners influenced the cleaners’ experiences of their work environmental problems related to the visibility, as social recognition or control. Furthermore, the concept of visibility is applied to analyse the cleaners’ experiences of invisibility at work.

### 1.2. Conceptual Reflections on Visibility as Social Recognition and Control

The concept of visibility was developed within the Social Sciences and is related to social interaction and social relationships, differentiation, power and social position, territoriality, ethnicity, and gender [12,13,14,15]. Furthermore, the concept of visibility has been used within studies of everyday life and working life, where different forms of visibility are practiced, changed, or negotiated within visibility regimes. A visibility regime is defined as the “systematic and routinary (*i.e.*, invisible) set-up of visibilities in contemporary social-technological complexes, as well as their contingent compositions” [13]. This means that a visibility regime can be explored for the effects of visibility and what they offer actors in different social settings and spaces. Visibility is not a social phenomenon with an essence, but rather an aspect of various social processes, it is the act of observing others and being observed. This dual action constitutes the subject within a social context of visibility [13]. On the one hand visibility can become a source of empowerment, when practiced as social recognition. This enhances social relationships and subjectivity. However, visibility can also result in disempowerment, when it is practiced as control. It may curb social relationships and subjectivity. This duality of visibility can serve to understand the dynamic relationship between individual experiences of visibility or invisibility. 

In relation to social position, visibility depends on a dominant vision within a social hierarchy. For instance, an ethnic minority group member, who is subjected to a dominant vision within a society of an ethnic majority group, can be rendered socially invisible. He or she cannot look back in social encounters as they are not seen. This can be explained by their subordinated position being excluded from the majority collective identity [13]. Furthermore group members from a minority or a low class can experience their invisibility as a lack of social recognition by a society in general or by actors in particular [13]. These examples of experiences of invisibility are not as clear cut as represented above because people interact within complex social situations, but nevertheless they illustrate how the actors’ experiences of visibility or invisibility are structured by their social position and social identity, such as ethnicity, in regimes of visibilities. Ethnicity in this article is understood as processes of creating and negotiating similarities and difference to other persons, groups, or communities through internal and externally identification, categorization, and classification [16]. We use the concept of visibility, as either control or social recognition, to analyse the different experiences of the cleaners in relation to visibility. For instance, we analyse why the cleaners’ descriptions of their psychosocial work environmental problems and experiences of a lack of visibility in their daily working routines were related to areas not being ready for cleaning by focusing on communication, social relations, and organisation at work. 

## 2. Methods

### 2.1. Data Collection and Study Population

The intervention was the second “Make a Difference” project initiated and run by the cleaning department of a Danish municipality and it was supported partly by the Ministry of Refugee, Immigration and Integration Affairs and partly by the Ministry of Occupation as well as The National Research Centre for the Working Environment (NRCWE). We evaluated the intervention, “Make a Difference”, in order to study the cleaners’ experiences of their psychosocial work environment, including cleaners’ influence or control of their work, knowledge of work environment health issues, social relations, and communication with managers and users as well as their well-being in general. 

#### 2.1.1. Qualitative Data Collection Approach

We chose a case study design [17] to investigate how suitable this type of intervention would be for cleaning staff with immigrant backgrounds and to establish whether it could serve as a “best practice” for comparable workplaces. In addition, the case study design was optimal to study the psychosocial work environment of the cleaners during their working routines and the daily social interactions within the group of colleagues or users at their workplace. Finally this design was fit to study the interactive processes among the different groups of employees and on-going social changes taking place influenced by the intervention or other changes. 

Our data sampling strategy consisted of mainly sampling qualitative data on particular experiences of the employees at the school within the context and work situation of this local workplace. However, we also used additional secondary material such as documents, reports, interviews and articles providing further information on the framework and context of these types of workplaces in general, Hence, the purpose of the data sampling strategy was multifold and the sampling process was divided into three main stages: before, during, and after the intervention: 

(1) Before the intervention we conducted interviews with the cleaners based on a semi-structured interview guide and the Copenhagen Psychosocial Questionnaire II (COPSOQ) using a digital voice recorder [11]. By using this method we could strengthen the qualitative specific data from the interviews with the uniform and scaled data from the COPSOC. Furthermore, we conducted individual semi-structured interviews with each of the employees to access sensitive information about various shared or individual views held by employees from different social backgrounds as well as employees holding different occupational positions. Interviewees with immigrant backgrounds were offered the assistance of a professional interpreter. In addition, the cleaning employees were provided with cameras to give them the opportunity to illustrate their working environment. The photo log, serving as a “statement” from the interviewee, was to be reflected upon during the second interview session. The photo log was a suitable tool for data sampling in cases where the interviewees were less articulate. And it proved successful since all the cleaning employees took photos relating to their work practices and volunteered to apply these during their second interview. 

(2) During the intervention we visited the workplace regularly to investigate the various dynamics and complex insider perspectives of the cleaners at the worksite. This was accomplished by observing and participating in training courses, team building, social events, and meetings. Field notes were taken according to the situation, employees, time, place, and types of interaction [18]. 

(3) After the intervention, interviews with the cleaning employees were repeated. Our questions invited the interviewees to explore and elaborate on his or her photos taken and presenting their experiences. In addition, the photos relating to work experiences before the intervention were discussed, comparing their experiences before the intervention to the present ones. The COPSOQ questionnaire was repeated. As a final question we asked each of the interviewee whether he or she would recommend the project or parts of it for other workplaces and their reasons for wanting to do so or not. In addition, we conducted three focus group interviews with teachers (one interview) and pupils (two interviews) to investigate whether they had been informed about the intervention and whether they had noticed any changes. A group of six teachers (one male and five female) was chosen to present general and specific views of the groups of teachers. The teachers were asked to recruit pupils for the focus group interviews and motivated pupils presenting the average pupil showed up, providing us with pupils of different grades (3 to 9) and ages (nine to fifteen). Both the teachers and the pupils were asked questions about the cleaning standard and the type of social relationship with the cleaners. Techniques used were listing and ranking of tasks and actions [19]. Photos taken by the cleaners were presented to discuss the cleaning services at the school. Finally, since the workplace was situated at a school, many social processes and developments occurred simultaneously that influenced the employees and their working routines. Therefore, we used secondary data; documents from the school or municipality, informing us of other changes, events, or interventions that occurred before, during, and after the intervention, to be able to assess, whether these could have influenced the opinions of the employees at the workplace [17]. In addition, we conducted interviews with cleaning managers at other schools where similar interventions had already been implemented. Data from these interviews provided additional information on how the organisation and management of the intervention could influence the outcomes. In total, 34 interviews were conducted with cleaners, managers, and school staff as well as three focus group interviews. 

Table 2 shows the profession, nationality, number of persons, and interviews as well as gender of the participants in the investigation. The group of cleaners between the ages of 25 and 60 years varied in terms of nationality; Danish, Thai, Turkish, former Yugoslavian, Filipino, and Romanian. The group of cleaners consisted of 10 females and four males. The working experiences of the cleaners ranged from one to fifteen years in the cleaning service at the school. Most of the cleaners with immigrant background had full-time employment contracts, some, however, only worked part-time due to their health. Two of the new cleaners were employed 5 hours a day which was the minimum hours for obtaining residency. The level of educational background varied from a very low level (Danes) to a relatively high level (Romanians). The participation of all the cleaners, teachers, and pupils in the interviews was voluntary and anonymous. 

**Table 2:** Study population divided into profession, nationality and gender.

Profession /Employment	Nationality	Persons (number of)	Interviews (per person)	Gender (Male/Female)
Cleaner	Danish	2	2	2 F
	Thai	2	2	2 F
	Turkish	1	2	1 M
	Former Yugoslavian	2	2	1 M/1 F
	Filipino	1	1	1 F
	Romanian	6	2	2 M/4 F
Manager	Danish	2	2	2 F
School leader	Danish	1	2	1 M
Caretaker	Danish	1	1	1 M
Teachers	Danish	6	1 (focus group)	1 M/5 F
Pupils	Danish	17	2 (focus group)	9 M/8 F
Total		41		16 M/ 25F

#### 2.1.2. Data Coding

**Figure 1:**
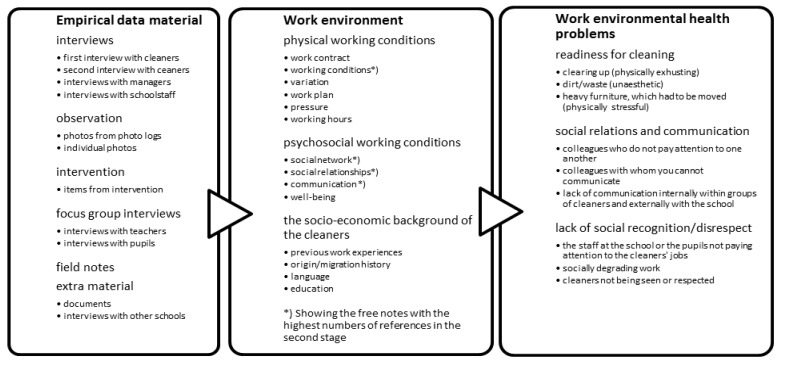
The coding process.

Interviews were transcribed, coded and analysed using in the qualitative data management program QSR NVivo 8, 2008. Figure 1 below shows the coding scheme [20] and the three basic stages of the coding process. 

(1) At the first stage the empirical data consisting of transcribed qualitative interviews with each employee was coded according to their occupational group, photos from the photo logs, focus group observation, field notes, school documents, and other material into *sources*.
(2) The interviews with employees were coded into *free notes* according to main categories from the questionnaires and additional questions arising from the interview sessions. Initially, each interviewer coded the texts according to their own codes and then conducted *intercoder agreement* selecting sets of codes most suitable for the further coding process [21]. When a photo from the photo logs or item from the intervention was referred to, this photo or item would be assigned a *memo*. The basic environmental health issues were condensed around working conditions, communication, working relationships, and social networks.
(3) At the third stage, the coded text, photo, or other items were further coded by a set of new *free notes*. The coded text was categorised by triangulating different sources of data [21]; documents from the intervention or the school, field notes from observation and participation, focus group interviews, or interviews with other school staff. These subcategories identified positive or negative statements of the psychosocial work environment of the employees within working conditions, communication, working relationships, and social networks. The subcategories were coded into three basic themes of “readiness for cleaning”, “social relations and communication” and “lack of social recognition and respect”. These three themes were selected for further analysis.

#### 2.1.3. Analysis

The themes “readiness for cleaning”, “social relations and communication”, and “lack of social recognition and respect” were analysed for how they were interrelated, and for the way the cleaners were organised internally within the cleaning group and externally at the school (the teachers and pupils) by looking at different types of communication, social contacts, and the different perspectives of the cleaners and teacher and pupils at the school. 

At first, perspectives of the cleaners presenting psychosocial health issues were analysed looking at social interactions, types of social relations and communication between the groups of cleaners internally in their subgroups and externally to other staff at the workplace, the school. This social interaction was examined at different locations and places at different times. Secondly, perspectives of the teachers and pupils were analysed to investigate how they experienced the social interaction and communication with the cleaners and the problem of “readiness for cleaning”. Thirdly the results of these two different perspectives related to the social interaction and communication between the different groups at different locations, times, and places at the school were analysed in order to identify where and how this problem occurred in the daily working routines and work organization at the school.

Finally, the cleaners’ opinions on whether the intervention had improved their psychosocial work environment were analysed. The observations and expression in the analysis were chosen to epitomise what we believe were central viewpoints presenting the themes and categories of the coding process. 

### 2.2. Validity

The results of the project evaluation, the coding, and analysis of the empirical data sample were presented and discussed both during and after the intervention on several occasions, including seminars with internal and external partners, presentation at the workplace with interviewed employees, managers and the vice principal. We presented the results of our coding and analysis in visual schemes and text citations in order to get feed-back from the interviewees. The triangulation of our interpretive framework by member checking helped us to validate and strengthen our interpretations and results [22]. Our results received positive responses and were considered constructive by researchers, employees, and employers, regarding the organisation and management of the psychosocial work environment interventions. Their comments and suggestions have helped to guide us in developing the arguments and conclusions presented in this article. But, owing to the lack of research in this field in general, it was difficult to compare our results to other research results at similar workplaces and groups of employees. Hence, we cannot extend the conclusions of this case study to other workplaces [17]. However, we can argue that the results and conclusions presented here are supported by other Danish studies on workplaces focusing on psychosocial work environmental health issues and the social positions of cleaning employees with an immigrant background, which are presented in the discussion. 

The cleaners with immigrant background have a lower and more insecure job position at the labour market than the Danish cleaners. The social position of the cleaners influenced the data collected concerning questions about the school staff. Being female, the researchers may also have caused a deference effect. This means that the responses of the interviewees especially with immigrant background to some extent can be biased to avoid offending the interviewer [23]. Furthermore, an expectancy effect of the researchers can have influenced the results as we visited the work place regularly in order to investigate changes taking place in relation to the intervention [23]. These two effects may have tainted the results so that the cleaners may have been overstating the positive effects of the intervention, and we may have over-interpreted the changes taking place. Thus, after the intervention, opinions on effects and changes were double checked in the interviews with the individual cleaners and in the discussions with the teachers and pupils at the focus groups interviews. 

### 2.3. The Case: “Make a Difference”

The intervention called “Make a Difference” was designed by the cleaning department of a municipality in North Zealand, Denmark, who was the service provider and employer of the cleaners at several schools. The aim of the intervention was to promote job adherence as well as to reduce sick leave and problems of recruitment of new cleaners. The schools were financed by the municipality, but they had the choice to either use the cleaning services of the municipally or buy services from a private company. Many schools had chosen the cleaning services of the municipality as this service administratively was easier to use. However, this school had been expressing dissatisfaction with the cleaning standards at the school for more than five years. The school was now considering hiring a private cleaning company. The municipality was at the time of the intervention effecting savings and the school was cutting back in teaching positions, which had raised the teachers’ stress level. 

The cleaning services, on the other hand, had had difficulties in recruiting new cleaners to work at the school. Instead, the manager had to use network recruitment, employing family members or friends of the cleaners’ at the school. In order to improve the critical conditions, the cleaning service section decided to implement the intervention “Make a Difference” at the school for an 8 month period between March and November 2009. The intervention consisted of different courses and events:

- staff meetings between the managers of the cleaning section, the cleaners and the school and the teachers
- posters with photos and names of the cleaners in the classrooms
- language courses in Danish
- courses in cleaning techniques
- social events and teambuilding

These elements were expected to improve the Danish language skills and knowledge of cleaning techniques among the immigrant cleaners as well as social interaction internally among the cleaners and externally to the school, the leaders, teacher, and pupils. These social, cultural, and technical skills were expected to raise the social position of and the status of the work of cleaning and the cleaners as employees and hence improve the well-being of the cleaners at work. However, not all planned activities were put into action, e.g., staff meetings with the school and teachers did not succeed, and the posters were either late or never put up.

## 3. Results

### 3.1. Social Relations and Communication at Work

The data showed that the cleaners’ work environment problems were related to specific tasks, areas, items, and social contact in their daily working routines and the following section examines the interaction of the cleaners and the school. In addition, it shows how the tasks and social contacts at the school were perceived by the different groups of cleaners and the school, the teachers, and pupils respectively. 

#### 3.1.1. Social Contacts among the Cleaners and their Contact with the Teachers and Pupils

During the cleaners’ daily working routines, they often met the teachers and the pupils in the school corridors, leaving or entering a classroom, or during breaks or after school in the schoolyard. These encounters were mainly brief, but sometimes informal and friendly verbal exchanges took place. However, situations with lacks of information by the teachers occurred. The information system at the school had been digitalized and most of the cleaners could either not operate the computer or did not understand enough of the Danish language to get the information needed. In addition, regularly social conflicts with the pupils occurred. This affected the cleaners’ well-being at the school. Below, the cleaners’ experiences of their social contacts are presented. 

#### 3.1.2. The Established Group of Cleaners

The established group of cleaners was differentiated in terms of work experience and ethnicity. This was reflected in their social contact to each other and to the teachers and the pupils. 

Some of the cleaners with more than 10 years of work experience at the school consisted of immigrants, mainly women, in their thirties to sixties, arriving in the 1980s or 1990s. In addition, the group also consisted of two female Danes and two middle aged men. They knew the school and the teachers very well and categorised the teachers as the “younger” or the “older” teachers and the cleaners felt that the younger teachers paid less attention to them than the older ones. This group of cleaners also knew the work routines and their roles in the division of work among all the cleaners at the school, and they often talked to the pupils. 

A female cleaner thinks of the school as her “home”: “*I like it here, it feels like home. I think things are working well with the teachers, the vice principal, and at the library. I think it is because I have worked here for so long*”. 

The group of cleaners seemed integrated as a group of colleagues, but social divisions could be observed. Internal social relationships within the established groups of cleaners and the new group of cleaners were expressed in different terms. Some cleaners from the established group of cleaners categorized the “new” cleaners as “foreigners” not socializing with their group of colleagues (“we”). Other cleaners from this established group resented this form of discrimination and expressed positive attitudes and relations to the new group. This division is presented in the following. Two of the cleaners’ emphasised the importance of socialising within the established group of cleaners: “*We all know each other and we like that we can sit here 2 or 3 people drinking coffee together on our breaks. And if others do not want to be here, they can just keep working*”. Another female cleaner explained: “*It is the foreigners who do not want to come down here*”. 

The group of cleaners shared their coffee breaks in the cleaners’ room in the cellar. Some, not everybody, resented the lack of social interaction with the group of new immigrant cleaners, who arrived in Denmark after 2007, mainly from Romania. They did not participate in the coffee breaks, but worked continuously as explained by the two female cleaners. However, another female cleaner expressed an opposite opinion: “*Some of the other cleaners do not like people from other countries, but I think that is silly. Some of us get along fine. But to avoid all these problems I have started working in the mornings*”. Another female cleaner confirmed having good relations with the group of new cleaners: “*I like it when the new cleaners get to know me. They are welcome to come and ask me if they need anything. And if I do not have it, I will try to get it for them. And if they need help, I will help them, happily*”. 

These different expressions illustrate that social divisions within the established group of cleaners and between the established group and the new group of cleaners existed. These internal social divisions were considered to have strained the social relations and communication and they influenced the psychosocial work environment of the cleaners before the intervention. 

Likewise, social relationships and communication to the teachers were expressed as strained by one female cleaner. 

She found the limited social contact with the teachers to be a problem as they did not notify the cleaners before meetings considering the limited timeslot for cleaning:

“*Normally the classes end at 2–2:15 pm. Last Friday I sat and waited for half an hour for the class to end so I could start cleaning it. When I got to the classroom the children were there waiting for me, telling me they had missed me because there was supposed to be a parent-teacher conference in 15 minutes and the classrooms had to be cleaned before that. But nobody gave me that information! And if I had known, I could have gotten a lot done in that half hour*”.

“*I got very annoyed because the teacher should have informed me about the parent- teacher conference. They see me every day but they just walk right past me. If they do not want to talk to me, they could just write the information on the blackboard*”.

Her negative attitudes were expressed through her strained relationship with the teachers, who never contacted or notified her. She experiences this invisibility as a lack of social recognition of herself as a colleague and of her cleaning. As mentioned in Section 3.1.1, the information system was digitalized. The cleaners had only received one hour of instruction in operating the computer and often important information was not received. Generally the cleaners were blamed by the teachers for not having cleaned the classrooms properly, and this lack of social contact to and invisibility by the teachers was a major source of frustration for the cleaners. 

The cleaners’ contact with the school was in several other ways limited by the lack of communication. A male cleaner describes an example of how he experienced the lack of communication and contact with the school at work:

“*Once I went to the vice principal’s office because I got the blame for the doors to the gym not being locked so people could just go in and use it. I told him, “You know, it was not me but the sports masters who forgot to lock the doors”. And the principal checked it out and now all the doors are locked*”.

When several users with different tasks in the same classrooms or halls coincided, and when a lack of communication existed (in this case between the teachers, cleaners, and the school leaders) confusion and frustration set in. Nobody knew who was responsible for the unlocked doors. However, communication between the teachers, cleaners, and school leaders helped to clarify the problem.

#### 3.1.3. The New Immigrant Cleaners

The group of new immigrant cleaners had between one and two years of work experience at the school. The group differed in relation to how well they spoke the Danish language and how well they knew the working routines at the school as well as the teachers and pupils. The newcomers’ social relationships internally within the group of cleaners and externally to the teacher and pupils reflected a situation requiring adjustment to overcome communication barriers. 

A cleaner, who had worked one year at the school said: “*Gradually they [the other cleaners] understand me better. We try to explain things to one another and I understand them better now, and we talk together a lot*”. 

The new immigrant cleaners’ social relationships with the *teachers* reflected a situation of mutual adjustment to overcome communication barriers at work as well. A female cleaner, who had worked one year at the school explained: “*The teachers are not like the pupils, there is a big difference. I can give you an example of that. Once, after a meeting, they [the teachers] did not tidy-up after themselves, so I did that for them. The next time the room was tidy. This means that they understood and respected my work and that is good*”. 

This explanation indicates a situation where the cleaner rather than talking to the teachers demonstrates herself what she expects from them. She does the clearing up that the teachers were responsible for and at the same time she achieves social recognition for her job, as a cleaner, who is taking the cleaning job seriously. Other cleaners in the new group had arranged with the teachers to put a notice on the door to the classroom, if they did not want their classroom cleaning at the usual time. They had found ways to communicate with the teachers, and they did not comment on the lack of contact by the teachers or being invisible, in spite of the reported communication barriers and the very limited contact to the teachers. But, as a female cleaner explained, visibility was very important for her work at the school: “*When the cleaning manager comes down to talk to us and knows all our names, and asks us how we are doing, take a look at us, appreciate us, and is responsive to us then I feel that I am noticed. And when you feel that you are noticed then you work with joy*”.

The new immigrants were more vulnerable in terms of social interaction with the older cleaners, teachers, and pupils. Their limited Danish skills posed a considerable challenge at work. 

### 3.2. Lack of Social Recognition and Respect

Both groups of cleaners with immigrant background expressed problems related to a lack of social recognition and respect, but towards different groups. Contact to the teachers was perceived as limited by some of the older cleaners: “*My cleaning is noticed, but I am not noticed as a person. The teachers walk right past us, but they never say “hello” or *“*hi” or anything. I think it would be nice if we could talk a little more with the teachers and if they would acknowledge us*”. Other problems related to communication between the cleaners and the pupils were expressed, and poor communication skills and a stigmatised ethnicity resulted sometimes in harassment by the pupils. A female cleaner, who had worked many years at the school, explained how she experienced this problem: “*They [the pupils] write on the blackboard that you are a cleaner, and they write dirty words, and sometimes they tease you when you are working by taking away your broom*”. A male cleaner from the new group of cleaners explained: “*The pupils do not think that cleaning is very important. Neither do they focus on, value, or pay attention to the cleaners*”.

The immigrant cleaners experienced different kinds of harassments, including racist insults. If teachers had left the classroom unlocked, frustration among the cleaners with immigrant background could be aggravated by the cleaners’ lack of communication with the teachers and the school managers. Teachers and school managers may not have been aware of the actions of the pupils, unless the cleaners reported them. The teachers were aware of one particular episode that had taken place, and after the cleaner complained to the school leaders the pupil was sanctioned. 

### 3.3. The Concept of “Readiness for Cleaning”

As part of the agreement between the cleaning department and the school, a classroom or any other common area should be left in such a state that it could be deemed “ready to be cleaned”. However, from the interviews with the cleaners and in their photo logs it was clear, that this often proved to be a general problem. Dirt or water was left on the floor, chairs were not placed at the tables, materials were destroyed, soaped paper balls were thrown to stick to the ceilings of bathrooms, floors were covered in resin, and doors were unlocked with pupils jumping on the tables. Nobody knew whether the teachers, the pupils, or the external users were to blame. But the problem left the cleaners with an extra work load and the impression that they had not done their jobs properly. This problem was reflected upon by the teachers and the pupils in the focus group interviews.

### 3.4. The Teachers

The group consisted of a team leader, class teachers, and a domestic science teacher. They reported that they often did not talk to the cleaners, but mainly greeted them when passing by. The teachers stated that:

“*the cleaners were a little bit invisible*”.

“*not all of them are speaking Danish*”.

“*the cleaners were more service workers than colleagues*”.

“*the cleaners have always been sort of isolated from the rest of the school staff*”.

The teachers only knew the names of a few cleaners from the old group and the name of only one of the newcomers. The communication between the teachers and the cleaners was more formal than informal and confined to specific areas and times at the school, such as at breaks or entering or leaving a classroom. 

The teachers’ statements in the focus group reflected their personal and professional positions according to how highly they prioritized cleaning tasks. But, in general the teachers did not prioritise the task of clearing up before leaving a classroom. This was explained by the fact that the teachers did not find sufficient time for all their duties. Instead, they prioritised teaching, meetings, and parental consultations. However, they mentioned the cleaning of the school as important for their well-being:

“*A clean school means that everything will work more efficiently*”.

“*In one word—it is well-being—that I like being at the school*”.

No cleaning meant the opposite of well-being:

“*It is like a snowball effect—the dirtier the school, the more of a mess the children will make*”.

“*I find it depressing to work in messy and dirty surroundings*”.

“*I think many of my colleagues have given up. They cannot be bothered to say anything anymore*”.

“*We should not get sick because of this [lack of cleaning]*”.

“*There should be a standard by which it is hygienically safe to work*”.

Even if the teachers considered cleaning at the school as important for their well-being, their low prioritisation of this task was discussed vividly. Some teachers expressed confusion about whose responsibility it was to prepare for cleaning: “*What I am struggling with is this: What is the responsibility of the pupils and what is the responsibility of the cleaners?”* Additionally they were concerned about the irresponsible behaviour of the pupils not fulfilling their duties as top boys or girls in the classes. Some teachers, however, were more concerned about the teachers’ roles: “*I think the biggest problem is that the teachers do not take the lead*”. A general consensus was reached as they all resented the harassment where pupils were spreading rumours of the immigrant cleaners stealing and sometimes insulting them. These incidents were considered to be unacceptable behaviour. 

### 3.5. The Pupils

The social contact between the pupils and the cleaners was very different from that of the teachers. The younger pupils from grades 3 to7 reported having more frequent contact with the cleaners than for example the secretaries or the principal. They spoke to the cleaners more frequently. This was also true for the pupils from grade 8 to 9 although not to the same extent as for the younger pupils. The pupils’ contact with the cleaners was often through face-to-face social encounters. Sometimes they would play football or play with the cleaners or contact particular cleaners to help them with their schoolwork. Their interactions were mainly with the “older” cleaners who spoke Danish. Pupils thought this social contact was positive. However, they mentioned language as a barrier for contact with some of the new immigrant cleaners. Their contact to the cleaners was a more direct informal interaction and it took place in different places, such as in the schoolyard, corridors, or in the classrooms before or after classes. 

The pupils, especially the youngest ones, prioritised learning to get the classrooms ready for cleaning higher than the teachers. For them, getting a classroom ready for cleaning was even more important than learning to become a good schoolmate. They mentioned that getting a classroom ready for cleaning was important for safety reasons (preventing the pupils from falling in the dirt). The older pupils prioritised *readiness for cleaning* lower, but never the less, higher than the teachers. The older pupils mentioned that getting a classroom ready for cleaning was associated with school work and they did not make any efforts with this task. 

### 3.6. Crossing Spaces and Tasks of the Cleaners, Teachers, Pupils, and Other Users

The experiences of the cleaners’ and the discussions of the teachers and the pupils revealed a problem with “readiness for cleaning” that was related to areas in which there were many different users at the school, both internal and external users. This resulted in confusion about their roles and responsibilities. Nobody knew exactly who was responsible or to blame, when rooms were left messy or damaged, or when things disappeared. This problem was reinforced by the lack of communication between the school, teachers, pupils, external users, and the cleaners. This confusion existed mainly where the different groups and their different tasks at the school crossed or intersected. The following model illustrates where the problem related to “readiness for cleaning” could be located:

**Figure 2:**
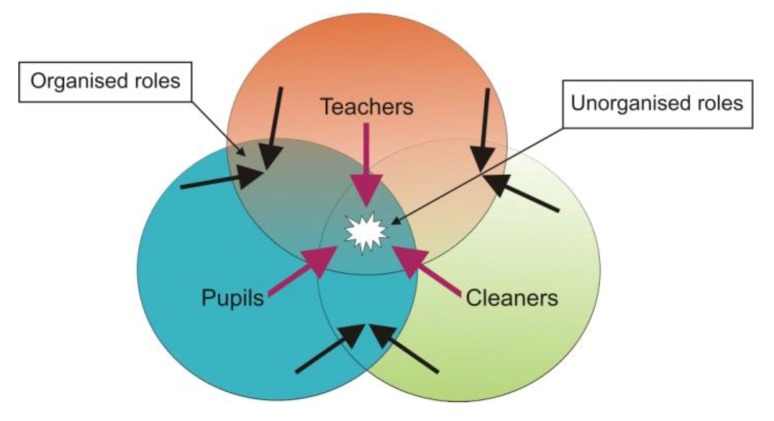
Crossing spaces and tasks among the cleaners, teachers, and pupils.

Figure 2 illustrates where tasks and places between the teachers, the pupils, and the cleaners crossed or intersected at certain times; during breaks, lunchtime, or after school and in certain spaces, such as in classrooms, the schoolyard, the gym, the corridors, *etc.* Normally teachers and pupils would interact in the classroom, or pupils and cleaners after class, or teachers and cleaners during breaks. In these encounters the parties seemed aware of their mutual responsibilities. The problems occurred when a third party was involved. 

As described in Section 3.4, the teachers’ social relations with the cleaners were constrained by limited contact. But, some of the older teachers did paid attention to the cleaners and their work, and these teachers knew the routines of getting a classroom ready for cleaning. However, as communication between the teachers and the cleaners had been digitalised, sometimes important information got lost. In addition, some of the teachers were confused about their own roles in areas where the teacher, pupils, and cleaners’ tasks crossed or intersected, for instance when they had left a classroom to pupils, and later the cleaners would arrive to clean. Some of the teachers did not know who was responsible for having messed up or vandalised a classroom. 

The pupils interacted with the cleaners in informal ways and especially the younger pupils had generally good contact with the cleaners. Moreover, the pupils could explain their own roles in relation to cleaning and were well aware of their duties in their classrooms. The pupils were less involved with the new immigrant cleaners and this relationship was characterised by poor communication. Problems occurred mainly when the pupils had left the classroom at breaks or after school. They too did not know who had removed their things: teachers, other pupils, external users, or the cleaners. 

The analysis illustrates that an organisation of working roles was needed. The lack of communication and clarified roles could be located between the school, concerning the teachers and the pupils on the one hand, and the cleaning staff on the other. An important question in this study was how the intervention affected the psychosocial work environment of the cleaners in relation to their influence on work, knowledge of work environment health issues, social relationships, and communication with managers and users at work, and their well-being in general. The next section will show how the cleaners experienced the differences that resulted from the intervention.

### 3.7. Differences Made by the Intervention

According to the cleaners, the intervention seemed to have clarified their roles vis-à-vis other school staff in places where tasks intersected and the division of roles were previously ambiguous.

A male cleaner explained this problem regarding cleaners’ roles at the school:

“*The allocation of work, not only among the cleaners, but also related to the principal, the teachers, and the school janitor, is important. Earlier, if all the chairs had not been hung from the tables, my colleagues would go into a classroom, hang up the chairs up, and sweep and wash the floors. But hanging up the chairs was not their job, and if they had only one hour to finish it all, they would never get it all done. I know that because it is written in my contract, but there are many cleaners who do not know that*”.

The cleaners did not always know which cleaning tasks at the school were theirs. But the intervention helped to clarify their work roles and responsibilities. 

In addition, the intervention gave the group of newcomers an opportunity to improve communication and social relationships with the older group of cleaners by participating in the language classes and in social events. Furthermore, they became more aware of their roles at work after the intervention. 

A male cleaner explained how he perceived the effects of the intervention:

“*We have learned not to do anything when teachers and pupils forget to put their chairs up on the tables since it is not our responsibility*”.

The cleaner expresses how, after being informed about the work rules, he has more control of his job by setting limits to avoid the extra tasks, which earlier were experienced as a strain on his health and general well-being. Indeed, some of the new immigrant cleaners explained that the intervention had improved their working conditions by clarifying their work roles. They knew when not to clean the classrooms, even if this would expose them to negative control and complaints from the teachers. Some mentioned improved and less messy classrooms after the intervention. 

A female cleaner explained that she did not know the other cleaners any better after the intervention, she mentioned however improved communication: “*I learned a lot from the Danish lessons. It is easier to talk to them [the other cleaners] now*”.

Being able to communicate in Danish with other colleagues was mentioned by most of the new cleaners as very positive, because language barriers generally were considered to have strained communication and social contact, not only within the group of new cleaners, but also by the group of established cleaners. One of the male cleaners from the group of established cleaners explained his experiences after the intervention:

“*Before, when the others sat down here and I sat up there, I would sit up there thinking ”are they talking about me now or what?” and that was not very smart, but then when I started to come down here I gradually felt more comfortable being here. At the same time my Danish improved and I could feel the difference and that has been a one of the successes of the intervention*”.

The results from the COPSOQ questionnaire showed a positive change in the cleaners’ psychosocial work environment related to social support, trust, and collaboration [11].

However, as the focus groups discussions showed, the intervention had limited effects on the social contact between the teachers, the pupils, and the cleaners. It did not improve or change the efforts of the teachers or the pupils in being responsible for getting a classroom ready for cleaning and they did not know about the intervention in general. Nor did the intervention increase the social contact between the cleaners, the teachers, and the pupils to such an extent that they were able to identify the new cleaners, their names, or the places where they were cleaning at the school. Only one of the new immigrant cleaners was noticed by the teachers, because she was cleaning in their staff room.

Thus the analysis suggests that the intervention, “Make a Difference”, helped especially the new cleaners to clarify their roles at work, enhancing their control of his or her job performance and reducing the work load. In addition, improved language skills improved social contact between the groups of new and old cleaners. This contact contributed to improving the general level of well-being at work. However, the intervention did not seem to affect the social contact between the cleaners and the school in more general terms. This could be explained by the failure of the managers to communicate the full scheme of the intervention, such as the staff meetings between the cleaning staff and the school staff, and with the posters not being made visible to everybody at the school. But, as the interviews with the cleaning managers from other schools, where the intervention was implemented, showed, a strong support from the school leaders was vital for the intervention to succeed. The cleaners and the cleaning managers expressed their disappointment that nobody from the school had participated in the staff meetings with the cleaners.

Initially, as described earlier, we wanted to investigate to what extend the ethnicity and social position of the cleaners influenced their experience of the psychosocial work environment and especially their perceptions of visibility as social recognition and control at work. This question will be discussed in the following.

## 4. Discussion

In the municipality, public cleaning had been outsourced for a number of years as a consequence of the restructuring of public spending and public management in general. However, at this school the municipality was the service provider and employer of the cleaners in the cleaning service. This meant that even though the cleaning staff and teachers were employed by different employers, they were paid by the same municipal employer. However, technically speaking the group of cleaners was no longer part of the school staff and they were not invited to attend either staff meetings or social events at the school for teachers or pupils. This was also reflected in the teachers’ categorisation of the cleaners as “service workers” not as colleagues and therefore considered as marginal employees at the school. 

The teachers did not attend the meetings in this intervention and they were not aware of the intervention at the school. This was interpreted by the cleaners as a lack of interest and recognition of them as employees. Furthermore, for a numbers of years the school had complained about the standard of cleaning in particular via the school’s newsletter. This negative opinion about the cleaning standard was also apparent in the teachers’ reflections of the cleaning standard at the school. Therefore, structural changes within public management and financial cuts in public spending, not only pressured the working pace of the cleaners and limited the cleaning standard, but it also influenced their social position at the school as “outsiders” [24]. This social position, combined with the negative attitudes and control in forms of complaints by the school of the cleaners’ cleaning services, can serve to explain why some cleaners who worked for a long time at the school felt a general lack of visibility as a lack of social recognition. They were no longer considered being a part of the school’s staff and readiness for cleaning was neither prioritised nor organised by the teachers or the school in general. Whether this lack of priority can be explained by a lack of resources or because the school in general considered these tasks to be a part of the “services” that they paid the cleaning service for, is an open question. 

Immigrant cleaners in cleaning services have increased significantly in recent years and the cleaning industry has become a segregated industry in which immigrants at some workplaces make up the majority of employees in traditional cleaning, thus often forming social networks based on ethnicity. Segregation occurs when employers and managers practice recruitment and employment of new employees based of their employees’ social networks. As Ejrnæs argues, while this recruitment strategy is practical and advantageous in many ways, e.g., maintaining a certain level of loyalty, it can reinforce a situation where cleaners with immigrant background become maintained in low positions [2]. When groups of employees are tied to strong social networks based on shared languages and backgrounds, conflicts between groups of employees based on internal prejudice occurs. These conflicts can spill over into organisational fragmentation and separatism. The more widespread network recruitment becomes within the cleaning industry, the more this industry becomes fragmented and segregated at the labour market [2]. This was also reflected in the group of cleaners at the school. Although two of the cleaners were native Danes, the majority had immigrant backgrounds. This social composition based on ethnicity was reflected by one of the established cleaners’ and the teachers’ characterisation of the cleaners as being mainly “foreigners”. The cleaners’ ethnicity had the effect of reinforcing their marginalised position in the social hierarchy of the school as “outsiders” and “foreigners”. 

The differences in social position and social divisions based on ethnicity, in terms of internal and external categorisations, within and between the established group of cleaners and new immigrant cleaners can help to explain why the newcomers did not experience the same lack of visibility as social recognition from the group of teachers as some of the established cleaners had expressed. The new group of immigrant cleaners had low expectations of their rights as employees compared to the established group of cleaners. The new cleaners did not express demands in the same way as the established group of cleaners when the classrooms were left messy. Their negative experiences were expressed towards pupils who verbally insulted the Romanian cleaners. This discrimination affected their well-being at the school. Furthermore, the new cleaners focused on the controlling aspect of visibility. They expressed concerns about dirt, vandalised items, *etc.* causing stress, as this would leave an impression of them not having done their jobs properly, putting their jobs at risk. Visibility of this group of workers was expressed differently than the established group of cleaners, where some emphasised the lack of social recognition by the teachers as a greater problem than the control of their work. However, the positive aspect of visibility as social recognition was expressed by one of the new cleaners, clearly testifying how important visibility as social recognition was for her well-being at work.

## 5. Conclusions

The intervention had limited effect on the main work problem of *readiness for cleaning* which could be located in poor communication between the cleaners on the one hand and the school on the other, particularly in areas where the three groups, the teachers (or other users), the pupils and the cleaners were crossing. As the intervention did not improve the contact between the cleaners and the school, this problem of a lack of communication and important school information, remained unresolved. However, the interventions did help the group of new immigrant cleaners to clarify their work roles and gaining control of their job performances, as they knew when not to clean the classrooms, and after the intervention instead of working overtime, they refused to clean the messy classrooms. Furthermore, after the intervention the communication between the two groups of cleaners improved.

Different explanations of the lack of visibility and social recognition experienced by the cleaners was presented and discussed, taking into consideration the structural settings, spatial separation of the cleaners’ workplace from that of their employers as well as social factors such as ethnicity, external and internal categorisations of “them” and “us”, and social position of the cleaners. 

The psychosocial work environment was experienced by the cleaners in general terms as a lack of visibility and social recognition by the school staff and pupils.

However, this lack of visibility as social recognition was highly influenced by the cleaners’ social position at work, which was experienced differently by the established groups of cleaners and the new group of cleaners. 

The newly arrived immigrants were not well integrated in the established group of cleaners. They were more isolated and had limited social contact with the other cleaners, teachers, and pupils in daily social encounters. Some of them, especially the Romanian cleaners, had experienced discrimination by the pupils, influencing their well-being at work. In addition, within the group of cleaners at the school, social boundaries existed, limiting social support by the older group of cleaners, when such assistance was needed. Furthermore, social barriers prevented collective action to raise the cleaners’ status at the school generally in a situation of difficult cleaning conditions.

Before the intervention, the new immigrant cleaners would clean the classrooms, even when they were not supposed to. They would not complain to the teachers or pupils if classrooms were left messy, since they were not in a position to complain, partly because of their low status at the school and partly because of poor Danish language skills. They experienced their visibility as disempowerment and control by the school. They emphasized that the dirt and damaged areas gave the impression of them not doing their jobs properly. Their insecure job position left them more vulnerable towards the negative criticism of the managers and the school staff. However, visibility as social recognition was expressed by a cleaner from the new group of cleaners in relation to the cleaning manager. Her experience clearly emphasised a sense of empowerment and subjectivity: “*When you feel that you are noticed then you work with joy*”. 

In the future employers should recognise that cleaners’ psychosocial work environment depends on the cleaners’ work experiences as well as their social position at work at the Danish labour market. This means that in order to improve communication and social contact at work, special efforts must be taken towards groups who are marginal at the labour market and unfamiliar with the language and safety rules concerning the psychosocial and physical aspects of cleaning. This can be done partly by job training and language courses (as included in this intervention) and partly by actively introducing, including, and engaging this group in staff meetings at their workplaces. This combination of inclusive measures would help new immigrant cleaners to clarify their roles in the organisation of workplaces and raise their status in the social hierarchy and social recognition as a group of employees rather than being perceived as “foreigners” and “service workers” who are mainly being controlled for the quality of their work. 

Part of the problem of lack of visibility and social recognition experienced by the cleaners in this study can be relevant for cleaners in other types of cleaning as well such as in offices, public areas, or other workplaces. In general, a more inclusive and positive attitude, based on social recognition of cleaners and their work tasks, may help to improve cleaners’ work environment including the psychosocial aspects of cleaning. 
